# Supplementary material for: Spotlight on New Hallmarks of Drug-Resistance towards Personalized Care for Epithelial Ovarian Cancer
Source: Cells. 2024 Mar 31;13(7):611. doi: 10.3390/cells13070611 (PMC11011744; doi:10.3390/cells13070611)
Supplement: Supplementary file 1 [file cells-13-00611-s001.zip › 28.03.24_Suppl_data_file1.pdf]

Supplementary data

Table S1. Current treatment regimens in platinum resistant EOC

	PLATINUM RESISTANT EOC	Ref.
**Cytotoxic drugs (preferred)**	Liposomal doxorubicin (PLD), gemcitabine, topotecan, etoposide, paclitaxel, cyclophosphamide, melphalan, vinorelbine, oxaliplatin:<br>- overall response rate (RR): 10-15%, progression-free survival (PFS): 3-4 m,<br>- overall survival (OS): 1 year	[2-3]
**Targeted therapies (preferred)**	Single agent Bevacizumab<br>Bevacizumab ± paclitaxel (only PFS benefit) → *EMA-FDA license*	[2-3]
**Hormone agents (preferred)**	Aromatase inhibitors (anastrozole, letrozole, exemestane), tamoxifen, leuprolide acetate, megestrol acetate	[2-3]
**Other therapies (special settings)**	Single-agent pembrolizumab for microsatellite instability-high (MSI-H) or tumor mutational burden-high (TMB-H) tumors ≥10 mutations/ megabase) without alternative options<br>Single agent pazopanib, trametinib (LGS) and Fulvestrant (LGS)<br>Dabrafenib + trametinib (for BRAF V600E-positive tumors)<br>Entrectinib and Selpercatinib (for NTRK and RET gene fusion positive tumors)<br>Mirvetuximab soravtansine-gynx/bevacizumab (for FRα-expressing tumors).	[3]
