# Supplementary material for: Spotlight on New Hallmarks of Drug-Resistance towards Personalized Care for Epithelial Ovarian Cancer
Source: Cells. 2024 Mar 31;13(7):611. doi: 10.3390/cells13070611 (PMC11011744; doi:10.3390/cells13070611)
Supplement: Supplementary file 1 [file cells-13-00611-s001.zip › 28.03.24_Suppl_data_file2.pdf]

Supplementary data

Table S2. Stem-cell markers used to characterize CSCs in EOC

CSC Marker	Biological function	Cell lines	Significance	Ref.
**CD24**	Cell surface sialo glycoprotein acting as adhesion molecule	Human HGS-OC EOC effusions and metastasis<br><br>CAOV3 cell line	- *in vitro* increased metastasis and chemoresistance<br>- poor outcome, relapse	[8]
**CD44**	Cell surface glycoprotein active in adhesions, migration, multi-ligand interactions (hyaluronic acid, osteopontin, collagens)	Human OC cells isolated from ascites and SKOV-3, OV90 and 3AO cell lines	- Predictor of relapse and worse survival in recurrent EOC	[8,22,32]
**CD117 (SCFR; c-Kit)**	Transmembrane tyrosine kinase receptor	Advanced HGSOC (SKOV-3, HEYA8, HO8910) cell lines)<br>OC xenografts	- Chemoresistance<br><br>- *in vivo*, paclitaxel increases CD177 expression<br>- higher tumorigenicity	[8,22,32]
**CD133 (prominin-1)**	Surface trans-membrane glycoprotein with a potential role in organizing plasma membrane topology	42 established OC cell lines and cells isolated from ascites	- Chemoresistance (higher Cisplatin IC_{50} in CD133+ cells)<br><br>- tumorigenicity vasculogenic ability indirect regulators (DNA binding protein ARID3B, miR-200a)	[8,22,32]
**ROR1**	Tyrosine-protein kinase transmembrane orphan receptor	Human HGSOC (OVCAR-3 cell line) and PDX samples	- higher tumorigenicity, EMT and chemoresistance.<br><br>- poor clinical outcome (overlap with ADH1)	[8,32]
**ALDH1**	Group of enzymes catalyzing the oxidation of intracellular aldehydes to their carboxylic acid forms	Human advanced HGSOC	poor survival, high tumorigenicity and	[8,20,38]

		(OVCAR-3 line) and PDX samples	chemoresistance (ADH1A expr.)	
**CD326/EPCAM**	Transmembrane glycoprotein driving Ca^{2+}-independent homotypic cell-cell adhesion			[8]
**CD 338/ABCG2**	Transporter of various molecules across extra- and intracellular membranes involved in chemoresistance		a possible role in chemoresistance	[8]
**CD 243/ABCB1/MDR 1**	Transporter of various molecules across extra- and intracellular membranes		a possible role in chemoresistance	[8]
**NANOG**	Transcription factor involved in self-renewal of undifferentiated embryonic stem cells	embryonic stem cells	negative prognostic factor	[8]
**OCT-4/POU5F1**	Transcription factor involved in self-renewal of undifferentiated embryonic stem cells	embryonic stem cells	negative prognostic factor	[8]
**SOX-2**	Transcription factor involved in self-renewal of undifferentiated embryonic stem cells	embryonic stem cells	negative prognostic factor	[8]
**MYC**	Oncogenic transcription factor; one of the reprogramming factors of induced pluripotent stem cells (iPSCs)	iPSCs	negative prognostic factor	[8]
**CD184/CXCR4**	α-chemokine receptor specific for SDF-1 overexpressed in CD133+ NYO-1 OC cells	CD133+ NYO-1 OC cells	a possible therapeutic target	[8]
**LIN28**[a]	RNA-binding protein; one of the reprogramming factors of iPSCs, overexpressed in CD44+ CD24+ EPCAM+ OC cell lines	iPSCs		[8]
**IL-17R**[a]	Receptor for IL-17, a pro-inflammatory cytokine produced by T-helper (Th17) cells, CD68+ macrophages and TAMs, overexpressed in CD133+ A2780 OC lines.	A2780 OC lines	a possible therapeutic target	[8]

a. LIN28 and IL-17R cannot be regarded as natural ovarian CSC markers, as they have not been used to prospectively isolate CSC-like cells but have been identified based on their higher expression levels in cell subsets isolated by different markers [13].
