# Supplementary material for: Spotlight on New Hallmarks of Drug-Resistance towards Personalized Care for Epithelial Ovarian Cancer
Source: Cells. 2024 Mar 31;13(7):611. doi: 10.3390/cells13070611 (PMC11011744; doi:10.3390/cells13070611)
Supplement: Supplementary file 1 [file cells-13-00611-s001.zip › 28.03.24_Suppl_data_file3.pdf]

Supplementary data

Table S3. Signaling pathways involved in drug resistance and potential targets for EOC.

Aberrant signaling pathway	Dysregulation and significance	Reversion mechanisms and (pre)clinical effects	Viable targets	Ref.
**Wnt/β-catenin**	- Normally: development of ovarian, fallopian tube stem cells<br>- **Dysregulation** promotes stemness, chemoresistance and poor OS<br>- Differences by histotype (rare in HGS)	- **preclinical use** of inhibitors, epigenetic inactivators, patient studies, cultures, animal models<br>- **reversion** of platinum-resistance in OC cell lines, mouse models	some pathway effectors	[7,22]
**MAPK/ERK**	- **MAPK** hyperactivation promotes proliferation, survival, metastasis, and chemoresistance<br>- **KRAS**: hyper-activated proliferative phenotype in LGSOC	- Endothelin-1 and IL-8-induced MAPK activation by EGFR/RAS signaling in SKOV-3 cells<br>- **chemo-resensitization** by reversion of inhibition	MAPK effectors	[25]
**PI3K/PTEN/AKT/ mTOR**	- Normally: cell growth, survival, motility, angiogenesis.<br>- **PI3K/AKT** hyperactivation promotes CSCs enrichment, maintenance, drug-resistance (cisplatin, paclitaxel)<br>- **hyperactivation** in 70% EOCs, pathogenesis of type I-II EOCs<br>- hyperexpression of activated AKT (pAKT): **poor survival** rate	-**preclinical** inhibitors, siRNA-AKT1 knockdown, AKT downregulation and PPM1D loss in cisplatin-resistant A2780 cells, and spheroids from SKOV3, HO8910 cell lines<br>- loss of CSC markers and **drug resensitization** (cisplatin, paclitaxel)	PI3K/PTEN/AKT effectors	[7,22,38]
**Notch**	- Normally: CSCs maintenance<br>- TCGA data: Notch 1-3-4/Jagged and downstream target genes (**NICD1-3**) **hyperexpression** promotes CSCs maintenance, EMT, metastasis, angiogenesis, drug-resistance<br>- **hyperactivation** in various EOCs<br>- **poor** clinical **outcomes** (Notch-3/ALDH1, Notch-1/NICD1)	- **preclinical** *in vitro* and *in vivo* inhibition of the Notch pathway, PDX models, suppression of Dll4 gene by Dll4-siRNA-conjugated nanoparticles<br>- suppressed tumor growth, angiogenesis, drug-resistance reversion in EOC	Notch 1-3-4 Jagged 1-2	[7,22]

**Hh**	- Normally: controls tissue polarity and stem cell maintenance.<br>- constitutive activation of Shh ligands or downstream effectors mutations (Patched, SMO, SUFU) or Gli transcription factors promote stemness, drug-resistance<br>- **hyperactivation** in 47% EOCs<br>- **poor** clinical **outcomes** (PTCH, Gli1)	- siRNA-mediated **Gli1 knockdown** in OC cell lines (SKOV3,OVCAR3, OVCA433)<br>- suppression of proliferation; **reversion** of cisplatin sensitivity	Hh effectors (PTCH, Gli)	[7,22]
**JAK/STAT 3**	- STAT3 hyperphosphorylation linked to CD24 CSC marker and promoter of EOC pathogenesis and metastasization;<br>- highly common **hyperactivation** (85% EOCs, 63% in HGSOC)<br>- phospho-STAT3 (pSTATe) nuclear expression: **poor survival** rates	- use of **JAK2 inhibitor** TG101209 alone in CD24+ cells and combined with cisplatin in in vivo mouse model; use of JAK2- inhibitor CYT38 plus paclitaxel in OC cell lines isolated from patient ascites<br>- reduced metastasis rate and better mice survival, higher cytotoxicity, **drug resistant reversion** and higher tumor shrinkage	JAK/STAT pathway effectors	[7,22,25]
**NF-KB**	- active promoter of CSCs maintenance, EOC invasiveness, metastasization via canonical (NEMO-dep.) and non-canonical (NEMO-indep.) manner<br>- CD44+/MyD88+ CSCs, by TLR4/MyD88/NFκB cascade, boosts proinflammatory TME and aggressive EOC phenotype<br>- ReIB **overexpression** in CSCs promotes carboplatin resistance<br>- **poor survival** rates	- in **preclinical** assays, use of NFκB inhibitor Eriocalyxin B; ReIB knock down<br>- **CSCs resensitization** to TNFα- and FasL-induced cell death; chemosensitization (carbo/cisplatin, paclitaxel sensitivity)	NF-KB mediators	[7,22]
**YAP-TEAD/Hippo**	- Normally: controls organ size<br>- YAP/TEAD complex involved in CSCs stemness, pluripotency, progression, chemoresistance<br>- TEAD1/3/4 **overexpression** fuels undifferentiated CSCs devoid of stem-like markers<br>- YAP **crosstalk** with other pathways (EGFR, RAS/MAPK, FOS/JUN, PI3K) for CSCs maintenance<br>- **poor survival** rates	- **YAP knock down** in A2780 OC cells; use of YAP inhibitors in OVCAR3 and OVCAR8 cell lines in vitro and OVCAR8 xenografts<br>- suppressed growth, migration, invasion, drug-resistance **reversion**; reduced tumor burden	YAP/TEAD complex	[7,22,39]

**Epigenetic changes**	- **Histone modifications:** chromatin bivalent state (H3K27me3 chromatin-permissive; H3K4me3 chromatin-repressive role)<br>- H3K27me3 and enhancer of zeste homologue 2 (EZH2) - mediated transcriptional silencing is typical in platinum-resistant EOC<br>- **histone deacetylation** (HDAC): SIRT1 as a player of chemoresistance in HGSOC by **crosstalk** with BRCA1, FOXO1 and EGFR<br>- **DNA methylation changes**: promoter silencing and drug-resistance.	- In cisplatin-resistant EOC cells, **reversion (demethylation)** of the DNA methylation changes in mismatch repair (MMR) genes (MLH1, MSH2) is likely to re-sensitize OC cells to following treatment	- DNA methyl-transferases (DNMT).<br><br>- Histone deacetylase (SIRT1)	[6,16,22]
