# Supplementary material for: Spotlight on New Hallmarks of Drug-Resistance towards Personalized Care for Epithelial Ovarian Cancer
Source: Cells. 2024 Mar 31;13(7):611. doi: 10.3390/cells13070611 (PMC11011744; doi:10.3390/cells13070611)
Supplement: Supplementary file 1 [file cells-13-00611-s001.zip › 28.03.24_Suppl_data_file4.pdf]

**Supplementary data**

**Table S4. New therapeutic avenues for drug resistant EOC**

| Drug class | Target/inhibitor | Phase | Findings | Ref. |
|---|---|---|---|---|
| TME-targeted therapies and immunotherapy | - CAF/TAM | Preclinical | - *Anti-CAFs* (MFAP5-mAb): reduced EOC proliferation, fibrosis | [22,25, 6] |
| | - PD-1: nivolumab | Phase 1b | - *AntiPD-1/PD-L1*: good safety profile<br>- *Nivolumab*: ORR 15%, PFS 3 m | |
| | - PD-L1 (avelumab) | Phase 1b | - *Avelumab*: ORR 13.6% | |
| | - PD-1 + FRα (pembrolizumab + mirvetuximab) | Phase 1b | - *Pembrolizumab + Mirvetuximab*: ORR 43%, PFS 5.2 m. | |
| | - PD-1: pembrolizumab | Phase 2 | - *Pembrolizumab* in MSI-H OC (KEYNOTE-158): ORR 33.3% | |
| | - PD-1 + microtubule (pembrolizumab + paclitaxel) | Phase 2 | -*Pembrolizumab + Paclitaxel* (synergistic effect): ORR 51%, PFS 6.7 m, OS 13.4 m | |
| | - PD-1 + VEGF + DNA damage (pembrolizumab + bevacizumab + cyclophosphamide) | Phase 2 | - *Pembrolizumab + Bevacizumab + Cyclophosphamide*: 6m-PFS 59% | |
| | - PD-1 + DNA cross-linker + nucleoside analogue (pembrolizumab + cisplatin + gemcitabine) | Phase 2 | - *Pembrolizumab + Cisplatin + Gemcitabine*: ORR 50%, PFS 5.4 m | |
| | - PD-1 + PARP (pembrolizumab + niraparib) | Phase 2 | - *Pembrolizumab + Niraparib*: 32% PD, 45% SD, 13% PR | |
| | - PD-1 + VEGF (nivolumab + bevacizumab) | Phase 2 | - *Nivolumab + Bevacizumab*: OR 16.7%, PFS 5.3 m | |
| ADCs | - DPEP3 (Tamrintanab pamozirine +/- budigalimab) | Phase 1 | - *Tamrintanab*: ORR 4%, SAEs 66%. | [6] |
| | - Mesothelin (Anetumab ravtansine + PLD) | Phase 1 b | - *Anetumab*: PR 52%, SD 33%, SAEs 95%. | |

| | | | | |
|---|---|---|---|---|
| | - NaPi2b (Lifastuzumab vedotin or PLD) | Phase 2 | - *Lifastuzumab vedotin or PLD*: PFS 5.3 m. | |
| | - FRα (mirvetuximab soravtansine) | Phase 3 | - *Mirvetuximab*: PFS 4 m, SAEs 26% | [6] |
| **Angiogenesis inhibitors +/- PARP inhibitors** | - VEGFR 1-2-3, PDGFR, c-Kit (pazopanib, cediranib) | Phase 2 | - *Pazopanib + Paclitaxel:* ORR 50%, PFS 6.3 m, OS 18.7 m.<br>- *Cediranib*: ORR 29%, PF 4.1 m, OS 11.9 m. | [6,25] |
| | - VEGFR 1-2 (aflibercept) | Phase 2 | - *Aflibercept*: ORR 12%, PFS 3.1 m. | |
| | - VEGFR 1-3, PDGFR, RET, FLT3, c-KIT, CSF1R (sunitinib) | Phase 2 | - *Sunitinib*: ORR 8.3%, PFS 2.3 m. | |
| | - VEGFR (Bevacizumab) | Phase 3 | - *Bevacizumab*: PFS 6.7 m, OS 16 m. | |
| | - VEGFR + PARP (Bevacizumab + Olaparib) | Phase 3 | - *Bevacizumab + Olaparib* in 1L EOC (PAOLA-1): PFS HR 0.31 and OS HR 0.60 in tBRCAm, PFS HR 0.33 and OS HR 0.62 in HRD-positive. | |
| **Wnt/β-catenin-targeted drugs** | -Disheveled (NSC668036, FJ9)<br>- Frizzled receptor (Ab)<br>- Porcupine (PORCN)<br>- β-catenin reverse transport (thiazolidinedione)<br>- β-catenin proteasome (Sulindac) | Preclinical *in vitro* results | *PDE10A gene knockout:*<br>- *In vitro*: anti- proliferative, anti-clonogenic potential in OC (SKOV3; OV-90) cell lines, suppression of major pathways (Wnt/β-catenin, RAS/MAPK, AKT) by RNA sequencing. | [1,7,22, 33,40] |
| | -Wnt/β-catenin + MAPK/AKT signaling (PDE10A inhibitors, e.g. Pf-2545920 and NSAID-derived MCI-030) | Preclinical *in vivo* results | *PDE10A gene knockout:*<br>- *In vivo:* decrease of tumorigenicity in mouse xenografts. | |
| | -WNT/β-catenin signaling (Ipafricept: recombinant fusion protein competing with FZD8 receptor for its ligand) | Phase 1b | *-Ipafricept (OMP54F28) + Carboplatin + Paclitaxel:* ORR 75.7%, PFS 10.3 m, OS 33 m, but bone toxicity at efficacy dose limited its further development. | |

| | | | | |
|---|---|---|---|---|
| **PI3K/PTEN/AKT/mTOR** | - PI3K/AKT pathway inhibitor overexpressed in PTEN loss<br>- AKT inhibitor MK-2206 | Phase 2 | - ORR 0%, SD 19 weeks | [6,22,26 33] |
| **Notch** | Notch-1 (inhibitors) Gamma-secretase inhibitors (GSI) | Preclinical | - *GSI siRNA transfection or DAPT treatment* may reverse drug resistance through impaired drug efflux or EMT, CSCs depletion, and apoptosis<br>- *Notch-1 inhibition/silencing* by miR-449a and miR-150 upregulation could reverse cisplatin- and paclitaxel- resistance by a pro-apoptotic effect. | [21,39] |
| **YAP-TEAD/Hippo targeting agents** | - YAP/TEAD (inhibitor verteporfin) | Preclinical | - *Verteporfin*: decreased proliferation, EMT, invasion in OVCAR3-OVCAR8 cell lines *in vitro* and tumor burden in OVCAR8 xenografts. | [22,39] |
| **Epigenetic inhibitors** | - DNMT inhibitors: Azacytidine | Phase 1/2 | - *5-Aza-dC + carboplatin*: efficacy (ORR 13%, PFS 3.7 m, OS 14 m). | [6,7,22] |
| | - Azacytidine (5-Aza-dC) | Phase 1 | - *5-Aza-dC + carboplatin + HDAC inhibitor* (NCT00529022): efficacy (SD 30%), safety | |
| | - DNMT inhibitors: Decitabine | Phase 2 (ongoing MITO 29 trial; *NCT 03467178*) | -Decitabine + Carboplatin vs treatment at physician choice (chemotherapy agents: PLD, paclitaxel, gemcitabine); primary endpoint (PFS). | |
| **Hh targeting-agents** | - SMO (sonidegib) | Preclinical | - *Sonidegib*: restores paclitaxel sensitivity, by decreasing MDR1. | [7,22] |
| | - SMO (vismodegib) | Phase 2 | - *Vismodegib*: negative results as maintenance therapy in EOC after 2nd - 3rd line complete remission. | |
| | - SMO (sonidegib) | Phase 1/1b | - *Sonidegib + paclitaxel*: ORR 22% | |

| | | | | |
|---|---|---|---|---|
| **DNA repair system doublets (HRR proficiency)** | - PARP + CTLA4 (Olaparib + Tremelimumab) | Phase 1 | - *Olaparib + Tremelimumab*: no SAEs | [6] |
| | - PARP (veliparib) | Phase 2 | - Veliparib: ORR 20%, safety | |
| | - PARP + VEGF (Olaparib + Cediranib) | Phase 2 | - *Olaparib + Cediranib:* ORR 20% (PR). | |
| | - PARP + VEGF (Olaparib + Cediranib or Paclitaxel) | Phase 2 | - *Olaparib + Cediranib/Paclitaxel*: PFS 5.7 m | |
| | - PARP (niraparib) | Phase 2 | - *Niraparib*: ORR 27% in BRCAm, 10% in HRD, 3% in HRR proficient | |
| | - PARP (rucaparib) | Phase 2 | - *Rucaparib*: ORR 25% | |
| | - PARP + VEGF (Olaparib + Cediranib) | Clinical - translational study | - *Olaparib + Cediranib* (translational): PFS (16 w): 50%; OS (1y): 64.8%. | |
| **Cell cycle checkpoint inhibitors** | - WEE1 G1-G2 (tyrosine kinase Adavosertib MK-1775)<br>- HSP90 (17- AAG, Ganetespib)<br>- ATR (M6620, perifosine, chlorambucil)<br>- bromodomain 4 (AZD 5153)<br>- CHK1/2 (praxerbitin) | Preclinical | - in vitro and in vivo studies with p53 targeting decreased proliferation, increased apoptosis, heightened cisplatin sensitivity in OCs with dysfunctional p53 | [6,25] |
| | - WEE1 G1-G2 (tyrosine kinase Adavosertib MK-1775) | Phase 1/2 | - *Adavosertib + Carboplatin* in p53-mutated refractory/resistant EOCs: efficacy (PFS, ORR) and good safety profile. | |
| | - TP53 (ganetespib) | Phase 1/2 | - *Ganetespib + Paclitaxel*: ORR 20%, AEs 30%, death 10% | |
| | PI3K/AKT (AKT inhibitor MK-2206, NCT 01283035) | Phase 2 | -MK2206: ORR 0%; SD 19 weeks in 1 patient. | |
| **Exosome-targeted therapies** | - Exosomes containing oncogenic promoters (inhibitors, e.g., GW4869, Dimethyl amiloride)<br>- Exosomal secretion (protein inhibitors) | Preclinical *in vitro* and *in vivo results* | - Use of exosome-inhibitors: induction of an antitumorigenic niche | [25] |

| | | | | |
|---|---|---|---|---|
| **HER2-inhibitors** | - HER2/HER3 (Pertuzumab) | Phase 2 | - *Gemcitabine +/- Pertuzumab*: ORR 13.8%, PFS 5.3 m. | [6] |
| | | Phase 3 | - *Pertuzumab + Topotecan/paclitaxel/gemcitabine*: PFS 4.3m; no OS differences. | |
| **Biomarker-driven targeted therapies** | - A molecular screening study for umbrella trial in platinum-resistant setting<br>- biomarkers: HRD and PD-L1 assessed during screening period | - biomarker-driven umbrella phase 2 trial | - efficacy endpoints: ORR 37%, m-PFS 4.76 m, m-OS: 15.51 m<br>- *In HRD+ subset:* promising efficacy and safety with Olaparib + cediranib/durvalumab<br>- *In HRD- subset*: promising efficacy and safety with ICI + standard CT, regardless of PD-L1 status. | [35-37] |
